# Cheap, Fast, Safe, Sustainable, and Positively Irresistible: The Future of Cationic Amphiphilic Disinfectant Development

**DOI:** 10.1021/acs.jmedchem.5c02054

**Published:** 2025-10-16

**Authors:** Makayla R. Hedges, Kevin P. C. Minbiole, Barry S. Selinsky, William M. Wuest

**Affiliations:** 1 Department of Chemistry, 1371 Emory University, Atlanta, Georgia 30322, United States; 2 Department of Chemistry and Biochemistry, 8210 Villanova University, Villanova, Pennsylvania 19085, United States

## Abstract

Disinfectants represent a crucial tool in killing infectious diseases and play a major role in protecting susceptible communities (hospitals and nursing homes). Inspired by nature, over the past 100 years, scientists have sought to recreate simplified antimicrobial peptides (AMPs) to serve these purposes. Quaternary ammonium compounds (QACs) have been mainstays in the industry, with benzalkonium chloride and didecyldimethylammonium chloride representing everyday disinfection agents. Through exhaustive investigations into alternative QAC structures, we have observed a “floor” in activity, with minimum inhibitory concentration (MIC) values of approximately 0.5 μM and no lower. Interestingly, the same value arises when one calculates the theoretical maximum in activity when considering the mechanism of action, aligning with the previously reported maximum inhibitory efficacy of AMPs. Herein, we encourage that the future development of novel disinfectants instead focus on five specific design elements: cost, time-kill, toxicity, sustainability, and resistance susceptibility.

## Graphical abstract

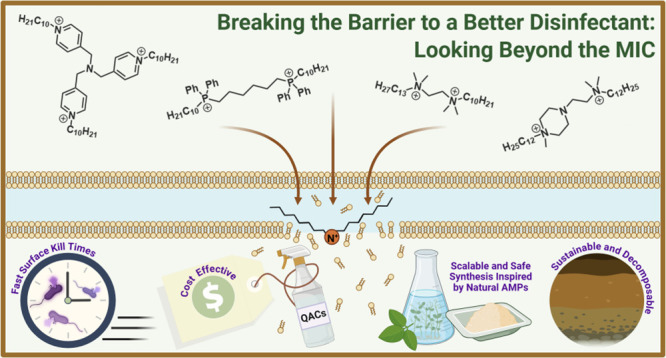

## Significance

This Perspective aims to reframe the current approach to the development of novel surface disinfectants, stepping away from a focus on potency, which seems to be approaching a mathematical limit due to cellular structure. A renewed focus on compound safety, minimal time-to-kill, inexpensive assembly, environmental friendliness, and overcoming bacterial resistance will bolster the utility of next generation quaternary ammonium compound (QAC) disinfectants.

## From Antimicrobial Peptides to Quaternary Ammonium Compounds

Disinfectant agents, though containing a diverse variety of chemical structures, are categorized by their broad-spectrum activity against a wide range of microorganisms, such as bacteria, viruses, and fungi. Hence, these make an ideal weapon against pathogenic bacteria present in susceptible communities.^1^ Cationic amphiphilic disinfectants have played a major role in this continuously developing field for 80 years or more, capitalizing on the net anionic composition of the bacterial cell membrane to initiate Coulombic interaction, which is followed by insertion of nonpolar tail(s), leading to membrane disruption and cell death.^2^ In many ways, these cationic compounds serve as mimics to what nature itself exploits for protection—antimicrobial peptides (AMPs)—in a simple but effective manner.^3^ AMPs are found in virtually all kingdoms, owing to their low energetic cost for synthesis and broad efficacy. AMPs are typically composed of both cationic and lipophilic amino acids displayed on opposite faces of the molecule to self-assemble with the phospholipids and/or itself to form aggregates that ultimately disrupt the bacterial membrane.

The study of antimicrobial peptides is extensive and there are many vital players in the field who have laid the foundation for groups like ours to apply their discoveries to the optimization of antimicrobial compounds. Zasloff and Lehrer were trailblazers in the discovery of novel AMPs and the understanding of their membrane-disruptive character.^4–10^ Researchers at the UK Health Security Agency like Hind and Sutton have also made significant advances in the understanding of the therapeutic efficacy of AMPs, as well as influential work with bolaampiphiles.^11–13^ The community continues to do impactful work through the implementation of modern techniques like Artificial Intelligence (A.I.) and Genome Mining. Notably, de la Fuente-Nunez et al. have used A.I. and machine learning to discover enigmatic antimicrobial peptides from even extinct species.^14–17^ We strongly recommend the reader to explore their cited work and reviews for broader understanding of the ideas presented herein.

## Their Synthetic Origin

Mimicking nature, synthetic chemists have replicated the general mechanism of action of these promising AMP therapeutics via quaternary ammonium compounds, or QACs. The first report of QACs appeared in 1915, when Jacobs and Heidelberger disclosed the preparation of antimicrobial derivatives of hexamethylene tetraamine, likely as a mixture of quaternary ammonium structures, possibly alongside formaldehyde.^18^ QACs gained significant acceptance in the 1930s and 1940s when benzalkonium chloride (BAC), a mixture of benzyldimethyl alkyl ammonium chlorides, was developed by Domagk^19^ and later marketed as Zephiran for use in surgical site preparation, leading to significantly improved clinical outcomes (Figure 1).^20^ Today’s disinfectant marketplace relies heavily on BAC, as well as other compounds including didecyldimethylammonium chloride (DDAC) and cetylpyridinium chloride (CPC). According to the EPA, in the USA alone QACs are produced or imported at more than 1 million pounds per year,^21^ only continuing to grow as the last quarter of the 20th century has led to the introduction of more novel analogs like octenidine and chlorhexidine. With enhanced activity arising from significantly different structures, these feature multiple cationic charges that are separated by an alkyl linker, creating bolaamphiphiles. Bolaamphiphiles are simply defined as bis-cationic structures where the charge is separated by a long alkyl chain. This optimized structure allows for strong activity against Gram-negative bacteria through improved outer membrane permeation (Figure 1, bottom left).^13,22^

**Figure 1:**
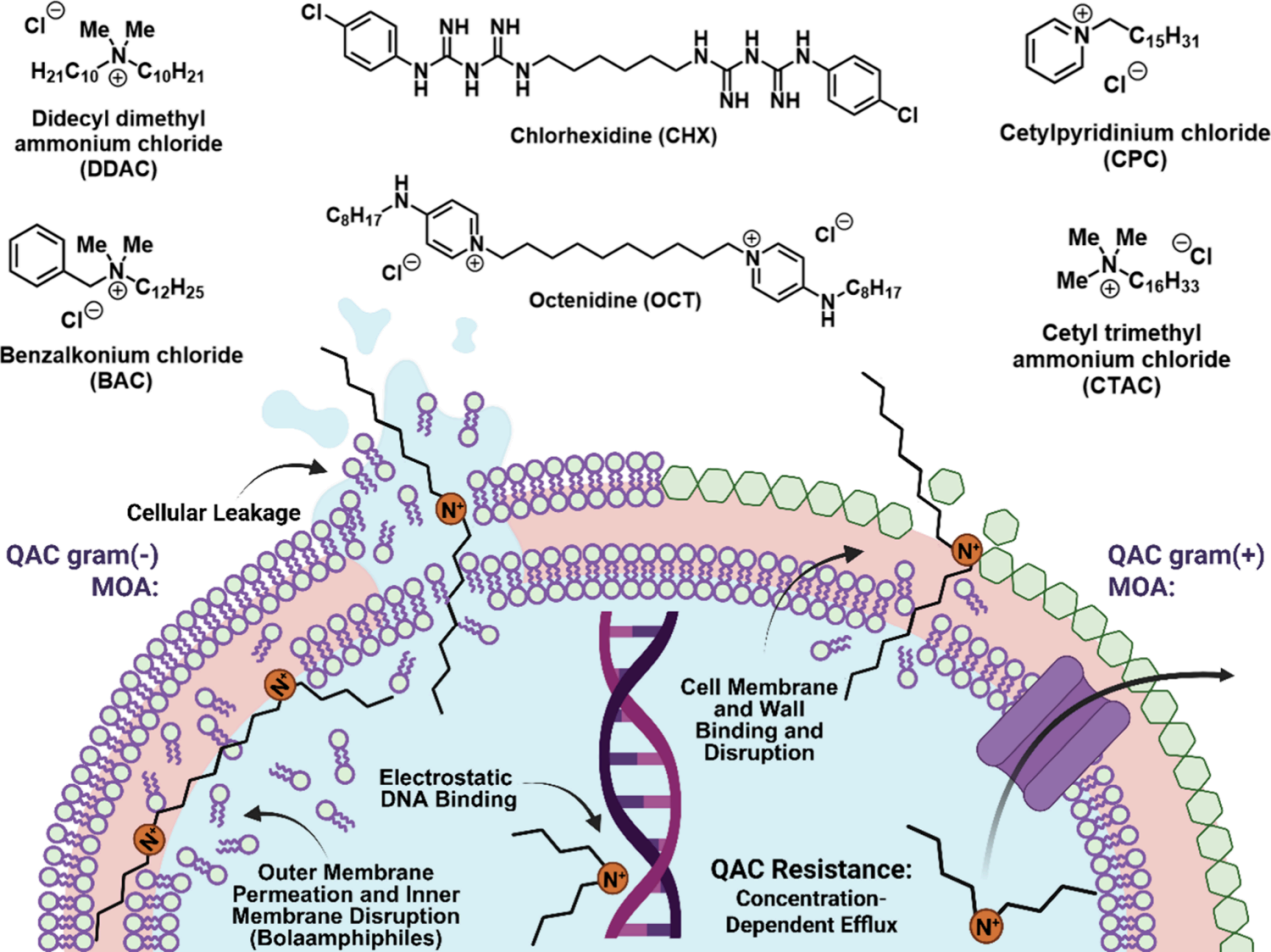
Mechanism of action and resistance of commercial QACs. Created in BioRender. Lab, W. (2025) https://BioRender.com/c10w89c.

## Inevitable Battle against Resistance

Given that QACs have a robust and nondiscriminant fundamental mode of action, it is somewhat surprising that bacterial resistance has been observed. Initial observations of QAC resistance were disclosed as far back as 1983 when plasmids in staphylococci were shown to confer resistance.^23,24^ Pioneering work by Skurray et al. in 1998 reported the elucidation of QacA, an efflux pump responsible for the expulsion of diamidine and biguanide disinfectants (Figure 1, bottom right).^25^ Despite many groups undertaking the microbiological exploration of these efflux pumps, and the negative transcriptional regulators that modulate their expression, little has been reported of their plasticity in recognizing substrates and whether these resistance mechanisms could be overcome with structural modifications. Work in our laboratories (K.P.C.M.; W.M.W.) has demonstrated that this resistance mechanism is somewhat paradoxical, seeing as for the mechanism to elicit its full effect a quaternary ammonium-type molecule must engage with a transcriptional regulator within the bacterium.^26^ Upon engagement, the regulator is then released allowing for the overexpression of efflux pumps that expel the active ingredient. At this time, it is still unclear if this effect is akin to a “treadmill” where the bacteria race to keep the concentration below the necessary threshold to kill by continuing pumping out the active agent, or if these pumps generate a pH gradient that might affect the net overall charge of the anionic phospholipid membrane and reduce the electrostatic potential. Nevertheless, our laboratories have shown that structural modifications to QAC architectures can overcome these barriers and result in either higher or lower affinities for specific membranes of bacteria.^27,28^ For example, recent work has shown that certain types of bolaamphiphiles tend to prefer aggregating at the inner membrane of Gram-negative bacteria, likely because of a preferred equilibrium disfavoring accumulation on the outer membrane.^29^ As a result, these compounds tend to be exceptionally potent and broaden the applications of disinfectants going forward. However, even with these recent discoveries, much still is left to do toward developing the “ideal” disinfectant that is broadly active across both susceptible and resistant Gram-positive and Gram-negative bacteria.

## Efforts by Our Groups

The idea of an “ideal” antibiotic has in turn led to a resurgence in the development of novel cationic amphiphilic disinfectants, with significant structural variation appearing in recent years. Work from our groups alone has resulted in nearly 50 publications and roughly 1000 distinct and novel cationic disinfectant structures.^27,28^ There has also been notable synthetic efforts by others like Vereshchagin and co-workers, specifically noting their expansion into the understanding of how linker flexibility of pyridinium-based bis-QACs plays a major role in activity.^30,31^ For more information into synthetic efforts of literature reported QACs, we direct the reader to the recent review by Fedorowicz and Sączewski.^32^ Through the Minbiole–Wuest collaboration, we have expanded to both quaternary phosphonium compounds (QPCs) as well as trivalent sulfur compounds (TSCs) to investigate the impact of changing the charge bearing atom.^29^ Interestingly, complete equivalence of activity was observed through a variety of structural pairs, exemplified by ^Me^P2P-12,12 ^33^ and 12(2)­12 which have perfectly analogous activity against a suite of Gram-negative and Gram-positive bacteria, as did the pair of ammonium and sulfonium species MP-18 and THT-18 (Figure 2).^29^ These and other similar observations suggest that the specific cationic center is not particularly influential in disinfectant bioactivity.

**Figure 2:**
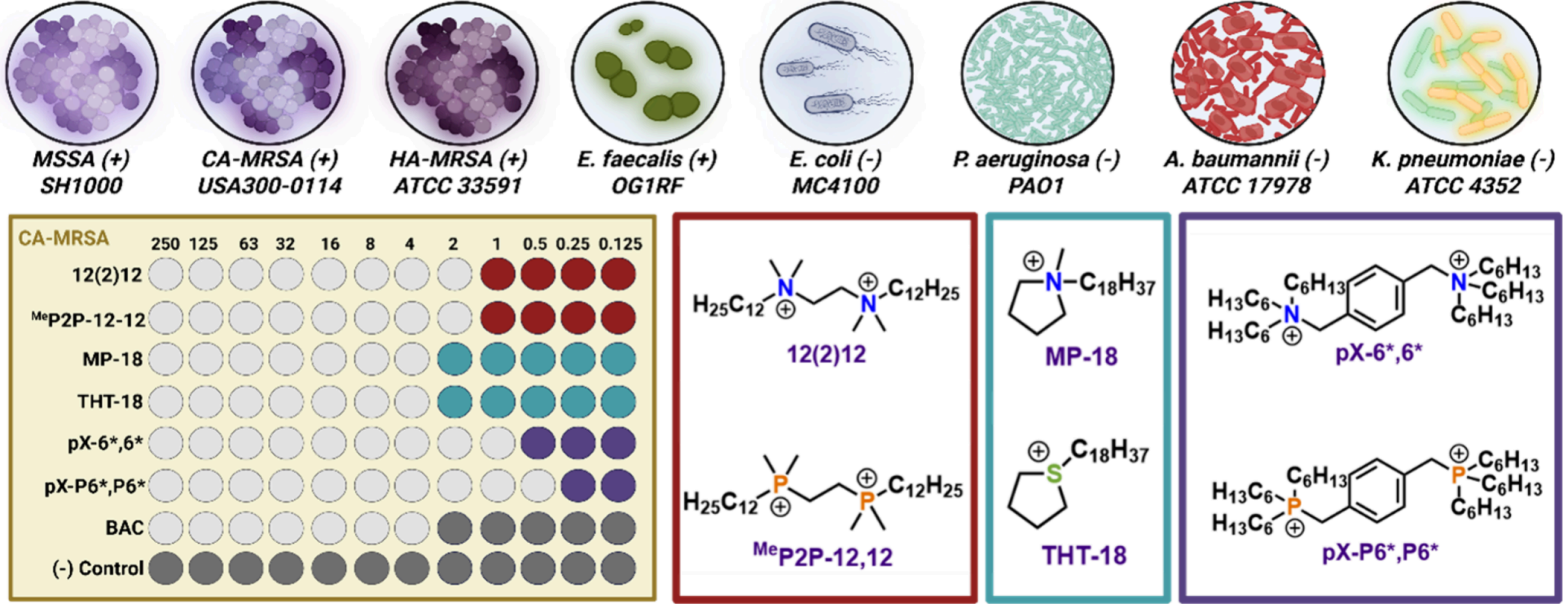
Representative MIC plate, 8 strain library available for Minbiole–Wuest QAC testing, and representative pairs that often show equipotent activity. Created in BioRender. Lab, W. (2025) https://BioRender.com/jwbkwlc.

Our own biotesting spanning over 1000 structures, against a panel of Gram-positive and Gram-negative bacteria, has led to an interesting statistical observation within those 5000+ generated data points. There is a repeated observation of similar minimum inhibitory concentration (MIC) values. We have observed that a wide variety of our most potent compounds, prepared and tested over a 15-year span, seem to display surprisingly uniform potency, despite a significant scope of structure that varies the number of cationic centers, the spacing between them, molecular architecture, and even element bearing the change. Our best compounds display MICs that are uniformly in the ∼0.5 μM region. In contrast, many of our modestly active compounds display MIC values that vary widely from ∼1 μM to 125 μM, with methicillin-susceptible *Staphylococcus aureus* often representing the lowest MIC value and Gram-negative strains like *Pseudomonas aeruginosa* the highest; however, improved disinfectants only display improved activity down to an observed “floor.” To illustrate this observation, Figure 3 presents a broad structural variety of cationic amphiphilic structures constructed in our laboratories, and virtually all demonstrate MIC values of ∼0.5–2 μM against all bacteria tested (typically a panel of susceptible and resistant *S. aureus*, as well as *Escherichia coli*, *Enterococcus faecalis*, *P. aeruginosa*, and oftentimes *Acinetobacter baumannii*.)^34–101^ The fact that these 13 compounds all deliver essentially the same bioactivity profile of quantified inhibition in MIC assays is suggestive to us of an overarching physical reality that warrants deeper consideration.

**Figure 3:**
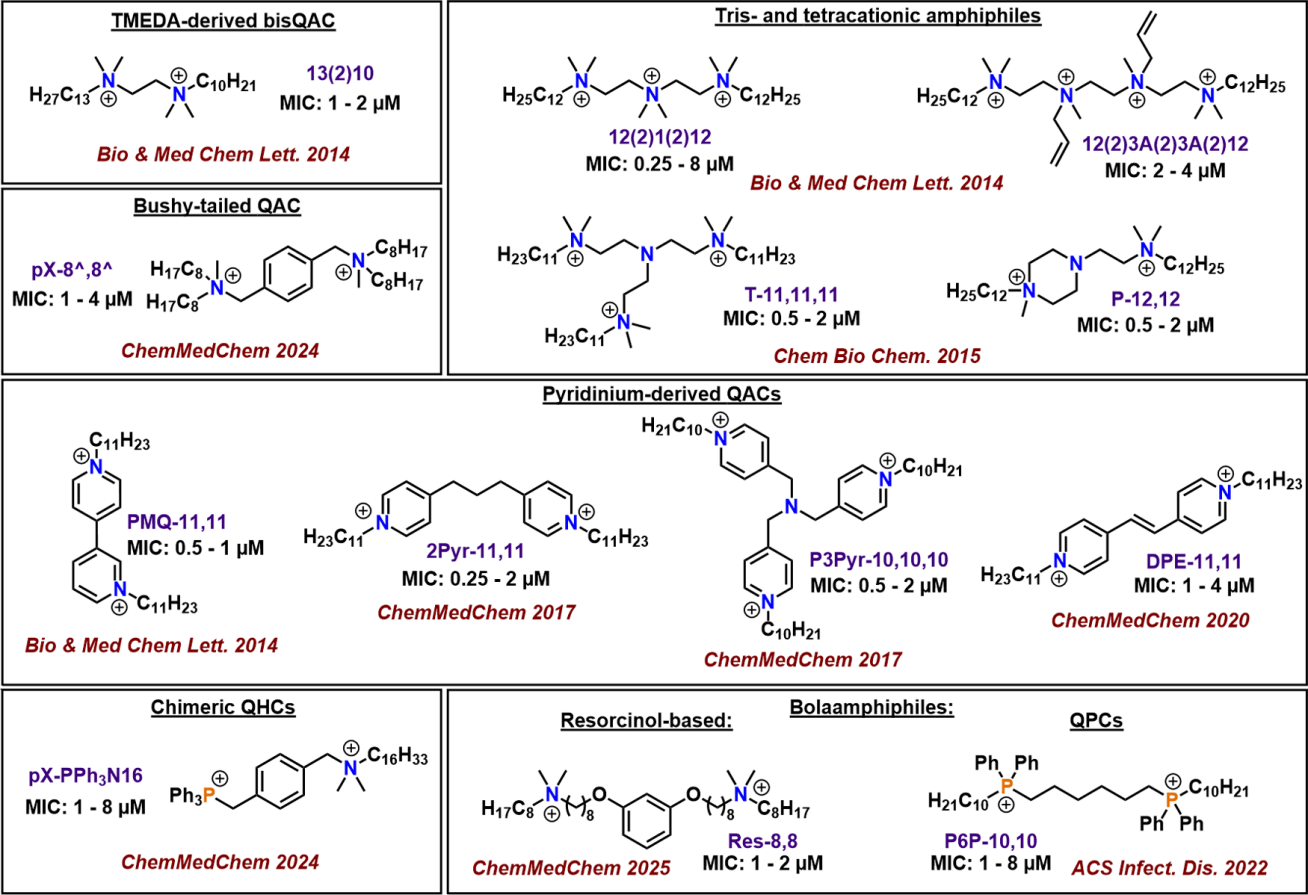
Quaternary ammonium/phosphorus/hybrid compounds prepared through Minbiole–Wuest collaboration tested against up to 8 bacterial strains.

Despite significant structural differences, antimicrobial peptides exhibit behaviors strikingly similar to QACs. AMPs are universally amphiphilic molecules with a positively charged polar region and an uncharged hydrophobic region; this is obtained through a variety of structures including helical, β-sheet, cyclical, globular, and other unique conformations.^42,43^ The MIC values for AMPs vary dramatically from one bacterial species to another, but the lowest reported value for naturally occurring AMPs is approximately 1 μM.^44,45^ Multiple attempts have been made to design AMPs with improved antimicrobial properties, and there has been some success in improving the selectivity of designed peptides toward specific species of bacteria, but no additional improvement in MIC values has been observed.^46–48^

## Taking Another Perspective

Taking together the observed “floors” of both the AMPs and QACs reported in literature, we began to wonder if there is a mathematical limit to the lysis of bacterial cells, based on the simple mechanics of membrane architecture.

The consensus opinion is that QACs kill bacterial cells by damaging the bacterial plasma membrane.^49^ Tischer et al.^50^ has provided this reasonable summary of the general mechanism of QAC action:

- Adsorption and penetration of QACs through the bacterial cell wall,
- Binding to components of the cell membrane,
- Disorganization of cell membranes leading to cell leakage,
- Intracellular degradation of proteins and nucleic acids,
- Lysis of cell wall components by release of autolytic enzymes.

To assess if this nonspecific model is consistent with measured MIC values, we can examine the nature of these measurements. In the determination of MICs for QACs, bacterial cell cultures are used with cell density of 5 × 10^6^ colony forming units (cells/mL). Each cell membrane contains approximately 10^7^ glycerophospholipids.^51^ In the simplest case, let us consider the Gram-positive pathogen *S. aureus* USA300 which only contains a single membrane. Their membranes are estimated to be comprised of 60% phosphatidylglycerol, 30% lysyl- phosphatidylglycerol, 4% diacylglycerol, and 2% cardiolipin.^52,53^ If QAC binding to lipids is driven by electrostatic interaction with anionic lipid headgroups, the zwitterionic lysyl- phosphatidylglycerol and neutral diacylglycerol should not represent a QAC binding site, leaving ∼60% of accessible lipids. If one works through the calculation: (cells/mL × lipids/cell × %anionic lipids) one would arrive at 3 × 10^13^ anionic lipids/mL.

How many QACs per cell are necessary for cell lysis? For antimicrobial peptides, the number of peptides needed to lyse a model membrane is estimated to be 200–2000 peptides per lipid vesicle.^50^ Wimley also does a detailed analysis of *in vivo* binding, indicating that there are 10–100 antimicrobial peptides bound per each lipid molecule amounting to as much as 10^9^ peptides bound per cell. Recent studies have demonstrated a ratio of 10:1 QACs/lipid in lysed cells, akin to AMPs. Using this multiplier arrives us at 3 × 10^14^ QAC molecules/mL needed to saturate the system. Finally, converting QAC molecules/mL to micromol/L arrives at a theoretical minimum MIC of 0.5 μM for Gram-positive *S. aureus* USA300. Again, this assumes that QACs have similar effectiveness to antimicrobial peptides and binds all available anionic lipids. This calculation is illustrated in eq 1.^54^

Biophysical studies describing the binding of QACs to phospholipid bilayers are extremely limited. The most relevant work describes the binding of cetylpyridinium chloride to phosphatidylcholine (PC) and phosphatidylcholine/phosphatidylglycerol (PC/PG; 8:2 ratio) bilayers. The free energy of binding and the equilibrium constant describing QAC partitioning into the membranes are approximately the same for the two lipid systems. Membranes containing PG have a greater binding enthalpy but lower binding entropy. Also, QACs are better able to effect dye leakage from PC vesicles than from PC/PG vesicles, suggesting that electrostatics is not a significant contributor to membrane binding.^51^ Membrane association of QACs is also dependent upon the ionic strength of the solution where the measurement is made.^51^ Finally, QACs have been shown to interact with bacterial cell walls, which would lower the concentration available to bind to the plasma membrane.^55^

1 $$\left(\frac{5\times 10^{6}\;cells}{1\;mL}\right)\left(\frac{10^{7}\;lipid\;memberanes}{1\,\;cell}\right)(0.6)=3\times 10^{13}\;lipid\;molecules\;per\;mL\;10\;QACs:1\;lipid\;to\;kill\;∴(\frac{3\times 10^{14}\;QAC\;molecules}{1\;mL})(\frac{1\;mol}{6.022\times 10^{23}\;molecules})(1\times 10^{6}\;\mu mol)=∼0.5\times 10^{-4}\;\frac{\mu mol}{mL}(\frac{1000\;mL}{1\;L})=0.5\;\mu M$$

One additional consideration is binding affinity. Many years ago, we (B.S.S.) measured the binding affinity of an antimicrobial lipid squalamine where the binding likely occurs at a spermidine substituent attached to a sterol ring. The binding of squalamine, which exists as a protonated polyamine in solution, to phosphatidylglycerol was determined to have a binding constant *K*_b_ = 1.2 μM. This number is also consistent with the best MIC values for QACs.

Considering this repeatedly observed and calculated physical limitation, we suggest a reframing of disinfectant development moving forward. We propose that there should be five key design principles in the development of novel disinfectants, which we will need to thwart bacterial resistance in future years. We have consolidated these principles into the title of this perspective (“Cheap, fast, safe, sustainable, irresistible”) and outline them here.

1. Cheap: Reduce the cost of production. To maximize the likelihood of industrial adoption of novel antibacterial structures, any proposed compound needs to be easily assembled at low cost. The fact that QAC and QPC structures are (by definition) salts allows for filtration-based purification strategies, which is inherently advantageous for scaleup. Further development of novel structures should bear in mind starting material accessibility, with attention paid to the changing availability of petroleum-based starting materials.
2. Fast: Minimize the time required for surface disinfection. While we have observed that minimum inhibitory concentrations face a mathematical limitation, there is ample evidence that physical dispersion of disinfectants can vary widely, and fast-dispersing compounds can translate into fast kill times on surfaces. Timed antimicrobial tests are the gold standard for assessment of activity but require significant investment of resources to develop suitable neutralizing solutions. Fortunately, we recently demonstrated^56^ that bubble pressure tensiometry can represent an inexpensive path for estimation of dispersion rates and can differentiate time-kill activity of compounds with identical MIC values. This phenomenon hints at supramolecular factors that may correspond to improved activity simply by modifying structures based on first-principles (i.e., noncovalent interactions, charges, and fundamental laws).
3. Safe: Minimize toxicity. For compounds to be widely adopted, toxicity must be minimized, as exposure is a risk starting from compound production, moving to utilization, and ultimately residing in waste management. Dermal and eye exposure represent the primary risks as most of these ingredients are used to treat everyday surfaces. We currently utilize hemolysis assays as a proxy assessment of cytotoxicity to eukaryotic membranes.^57^ Recent work has also exposed reproductive risks and exposure in blood samples, but future work is needed to bear out these outcomes.^58^
4. Sustainable: Reduce environmental waste. Environmental concerns also play a significant role when deciding to move a new active forward. One path toward good stewardship of the earth’s resources is to design purposefully unstable structures, termed “soft”^59^ or “self-destruct” to minimize the long-lasting impact in nature and reduce environmental exposure. Our group and others have developed several analogs wherein long alkyl chains are modified to contain cleavable ester and amide moieties, ensuring a site of decomposition in environments of modified pH (e.g., soil). An aforementioned second path to consider is leveraging sustainable and benign starting materials that can be sourced cheaply and reliably, for example, from chemical feedstocks or potentially waste streams from other commodity chemical production.
5. (Positively) Irresistible: Overcome bacterial resistance mechanisms. Bacterial resistance is an inevitable fact in the development of any antimicrobial compound. Structure activity relationships have, over time, identified key privileged structures that can elude bacterial resistance. This can rely on the continuous development of novel structures, as well as structural classes that have demonstrated minimal resistance behaviors (multicationic species, bolaamphiphiles) based on inherent mechanisms. It can also involve the development of the aforementioned “self-destruct” molecules to limit the time that bacteria are exposed to the actives in the environment. Continual chemical innovation and proper stewardship will be critical for prolonging the effectiveness of these vital disinfectants going forward.

The above analysis is perhaps overly simplistic, as the real world of pathogen inhibition brings with it additional concerns. Among these are aggregation behavior, the heterogeneity of cell surfaces, bacterial variation, and the presence of nonbacterial pathogens. First, the aggregation behavior of amphiphiles into micelles and other supermolecular structures presents a reservoir which diminishes the actual concentrations of these compounds and may in fact represent an alternate mode of activity of bacterial inhibition. Second, there exist other distractors for binding, even in the most simplistic of MIC analyses, including cell surface proteins, extracellular matrices, and perhaps even the surface of the testing vessel itself. Third, one must acknowledge the tremendous variability among bacterial species and strains; a simple assessment of percentage of membrane components could be no better than an estimate or average. And finally, bacteria are not the only human pathogens; development of a true disinfectant should neutralize fungal and other pathogenic species.

Despite these significant limitations toward any mathematical analysis of molecular behaviors, an overriding theme remains clear: roughly 1000 disinfectant QACs and related species have, in our hands, shown a “floor” of activity. A pivot to alternative strategies for cationic disinfectant development would breathe new life into this crucial upstream mode of health protection.

## Abbreviations Used

A.I.: artificial intelligence
AMPs: antimicrobial peptides
BAC: benzalkonium chloride
CPC: cetylpyridinium chloride
DDAC: didecyldimethylammonium chloride
EPA: U.S. Environmental Protection Agency
MIC: minimum inhibitory concentration
QACs: quaternary ammonium compounds
PC: phosphatidylcholine

## References

[1] Denyer S. P., Stewart G. S. A. B. (1998). Mechanisms of action of disinfectants. International biodeterioration & biodegradation.

[2] Gilbert P., Moore L. E. (2005). Cationic antiseptics: diversity of
action under a common
epithet. Journal of applied microbiology.

[3] Bahar A. A., Ren D. (2013). Antimicrobial peptides. Pharmaceuticals.

[4] Zasloff M. (1987). Magainins,
a class of antimicrobial peptides from Xenopus skin: isolation, characterization
of two active forms, and partial cDNA sequence of a precursor. Proc. Natl. Acad. Sci. U.S.A..

[5] Zasloff M. (2002). Antimicrobial
peptides of multicellular organisms. Nature.

[6] Lazzaro B. P., Zasloff M., Rolff J. (2020). Antimicrobial
peptides: Application
informed by evolution. Science.

[7] Hancock R. E. W., Lehrer R. (1998). Cationic peptides: a new source of
antibiotics. Trends Biotechnol..

[8] Ganz T., Selsted M. E., Szklarek D., Harwig S. S., Daher K., Bainton D. F., Lehrer R. I. (1985). Defensins.
Natural peptide antibiotics
of human neutrophils.. J. Clin. Invest..

[9] Lehrer R. I., Lichtenstein A. K., Ganz T. (1993). Defensins: antimicrobial and cytotoxic
peptides of mammalian cells. Annu. Rev. Immunol..

[10] Lehrer R. I., Ganz T. (1999). Antimicrobial peptides
in mammalian and insect host defense. Current
Opinion in Immunology.

[11] Fields F. R., Manzo G., Hind C. K., Janardhanan J., Foik I. P., Carmo Silva P. D., Balsara R. D., Clifford M., Vu H. M., Ross J. N., Kalwajtys V. R., Gonzalez A. J., Bui T. T., Ploplis V. A., Castellino F. J., Siryaporn A., Chang M., Sutton J. M., Mason A. J., Lee S. (2020). Synthetic Antimicrobial Peptide Tuning
Permits Membrane Disruption
and Interpeptide Synergy. ACS Pharmacol. Transl.
Sci..

[12] Manzo G., Hind C. K., Ferguson P. M., Amison R. T., Hodgson-Casson A. C., Ciazynska K. A., Weller B. J., Clarke M., Lam C., Man R. C. H., Shaughnessy B. G. O., Clifford M., Bui T. T., Drake A. F., Atkinson R. A., Lam J. K. W., Pitchford S. C., Page C. P., Phoenix D. A., Lorenz C. D., Sutton J. M., Mason A. J. (2020). A pleurocidin analogue with greater conformational
flexibility, enhanced antimicrobial potency and in vivo therapeutic
efficacy. Commun. Biol..

[13] Di
Blasio S., Clarke M., Hind C. K., Asai M., Laurence L., Benvenuti A., Hassan M., Semenya D., Man D. K. W., Horrocks V., Manzo G., Van Der
Lith S., Lam C., Gentile E., Annette C., Bosse J., Li Y., Panaretou B., Langford P. R., Robertson B. D., Lam J. K. W., Sutton J. M., McArthur M., Mason A. J., Castanheira M. (2023). Bolaamphiphile Analogues of 12-bis-THA Cl 2 Are Potent
Antimicrobial Therapeutics with Distinct Mechanisms of Action against
Bacterial, Mycobacterial, and Fungal Pathogens. Msphere.

[14] Wan F., Wong F., Collins J. J., de laFuente-Nunez C. (2024). Machine learning
for antimicrobial peptide identification and design. Nature reviews bioengineering.

[15] Hao Y., Wang J., de la Fuente-Nunez C., Franco O. L. (2022). Editorial: Antimicrobial
Peptides: Molecular Design, Structure-Function Relationship, and Biosynthesis
Optimization. Front Microbiol..

[16] Torres M. D. T., Brooks E. F., Cesaro A., Sberro H., Gill M. O., Nicolaou C., Bhatt A. S., de la Fuente-Nunez C. (2024). Mining human
microbiomes reveals an untapped source of peptide antibiotics. Cell.

[17] Santos-Júnior C. D., Torres M. D. T., Duan Y., Rodríguez del Río Á., Schmidt T. S. B., Chong H., Fullam A., Kuhn M., Zhu C., Houseman A., Somborski J., Vines A., Zhao X. M., Bork P., Huerta-Cepas J., de la Fuente-Nunez C., Coelho L. P. (2024). Discovery of antimicrobial
peptides
in the global microbiome with machine learning. Cell.

[18] Jacobs W. A., Heidelberger M. (1915). On a new group of bactericidal substances obtained
from hexamethylenetetramine. Proc. Natl. Acad.
Sci. U. S. A..

[19] Domagk G. (1935). Eine neue
klasse von desinfektionsmitteln. DMW-Deutsche
Medizinische Wochenschrift.

[20] Rahn O., Van Eseltine W. P. (1947). Quaternary
ammonium compounds. Annu. Rev. Microbiol..

[21] United States Environmental Protection Agency (EPA) Information about Chemical Data Reporting. https://www.epa.gov/chemical-data-reporting/basic-information-about-chemical-data-reporting#what.

[22] Bezold E. L., Young A. L., Jaworski C. J., Minbiole K. P., Sanchez C. A., Wuest W. M. (2025). Bisphosphonium Amphiphiles
Yield Insights into Gram-Negative
Bacterial Disinfectant Resistance and Cell Membrane Interactions. ACS Omega.

[23] Archer G. L., Johnston J. L. (1983). Self-transmissible
plasmids in staphylococci that encode
resistance to aminoglycosides. Antimicrob. Agents
Chemother..

[24] McDonnell R. W., Sweeney H. M., Cohen S. (1983). Conjugational transfer of gentamicin
resistance plasmids intra-and interspecifically in Staphylococcus
aureus and Staphylococcus epidermidis. Antimicrob.
Agents Chemother..

[25] Grkovic S., Brown M. H., Roberts N. J., Paulsen I. T., Skurray R. A. (1998). QacR is
a repressor protein that regulates expression of the Staphylococcus
aureus multidrug efflux pump QacA. J. Biol.
Chem..

[26] Jennings M. C., Minbiole K. P., Wuest W. M. (2015). Quaternary ammonium
compounds: an
antimicrobial mainstay and platform for innovation to address bacterial
resistance. ACS infectious diseases.

[27] Minbiole K. P., Jennings M. C., Ator L. E., Black J. W., Grenier M. C., LaDow J. E., Caran K. L., Seifert K., Wuest W. M. (2016). From antimicrobial
activity to mechanism of resistance: the multifaceted role of simple
quaternary ammonium compounds in bacterial eradication. Tetrahedron.

[28] Morrison K. R., Allen R. A., Minbiole K. P., Wuest W. M. (2019). More QACs, more
questions: Recent advances in structure activity relationships and
hurdles in understanding resistance mechanisms. Tetrahedron letters.

[29] Feliciano J. A., Leitgeb A. J., Schrank C. L., Allen R. A., Minbiole K. P. C., Wuest W. M., Carden R. G. (2021). Trivalent sulfonium
compounds (TSCs):
Tetrahydrothiophene-based amphiphiles exhibit similar antimicrobial
activity to analogous ammonium-based amphiphiles. Bioorganic & medicinal chemistry letters.

[30] Frolov N. A., Tyutin A. A., Tyurina A. N., Seferyan M. A., Detusheva E. V., Son E., Saverina E. A., Vereshchagin A. N. (2025). Expanding the Variety of Pyridinium-Based
Bis-QACs with Antimicrobial Properties: Investigation into Linker
Structure-Activity Correlation. ChemMedChem.

[31] Vereshchagin A. N., Frolov N. A., Egorova K. S., Seitkalieva M. M., Ananikov V. P. (2021). Quaternary Ammonium Compounds (QACs)
and Ionic Liquids
(ILs) as Biocides: From Simple Antiseptics to Tunable Antimicrobials. International journal of molecular sciences.

[32] Fedorowicz J., Sączewski J. (2024). Advances in
the Synthesis of Biologically Active Quaternary
Ammonium Compounds. Int. J. Mol. Sci..

[33] Thierer L. M., Petersen A. A., Michaud M. E., Sanchez C. A., Brayton S. R., Wuest W. M., Minbiole K. P. C. (2023). Atom
economical QPCs: phenyl-free
biscationic quaternary phosphonium compounds as potent disinfectants. ACS Infectious Diseases.

[34] Black J. W., Jennings M. C., Azarewicz J., Paniak T. J., Grenier M. C., Wuest W. M., Minbiole K. P. C. (2014). TMEDA-derived
biscationic amphiphiles:
An economical preparation of potent antibacterial agents. Bioorg. Med. Chem. Lett..

[35] Paniak T. J., Jennings M. C., Shanahan P. C., Joyce M. D., Santiago C. N., Wuest W. M., Minbiole K. P. C. (2014). The
antimicrobial activity of mono-,
bis-, tris-, and tetracationic amphiphiles derived from simple polyamine
platforms. Bioorg. Med. Chem. Lett..

[36] Ator L. E., Jennings M. C., McGettigan A. R., Paul J. J., Wuest W. M., Minbiole K. P. C. (2014). Beyond paraquats: Dialkyl 3, 3′-and 3, 4′-bipyridinium
amphiphiles as antibacterial agents. Bioorganic
& medicinal chemistry letters.

[37] Mitchell M. A., Iannetta A. A., Jennings M. C., Fletcher M. H., Wuest W. M., Minbiole K. P. C. (2015). Scaffold-Hopping
of Multicationic Amphiphiles Yields
Three New Classes of Antimicrobials. ChemBioChem..

[38] Leatherbury M. S., Thierer L. M., Sanchez C. A., Vargas-Cuebas G. G., Petersen A. A., Amoo L. E., Bezold E. L., Washington K. C., Mistrot M. B., Zdilla M. J., Wuest W. M., Minbiole K. P. C. (2024). Chimeric
amphiphilic disinfectants: quaternary ammonium/quaternary phosphonium
hybrid structures. ChemMedChem.

[39] Al-Khalifa S. E., Jennings M. C., Wuest W. M., Minbiole K. P. C. (2017). The Development
of Next-Generation Pyridinium-Based multiQAC Antiseptics. ChemMedChem..

[40] Toles Z. E., Thierer L. M., Wu A., Bezold E. L., Rachii D., Sanchez C. A., Vargas-Cuebas G. G., Keller T. M., Carroll P. J., Wuest W. M., Minbiole K. P. C. (2024). Bushy-Tailed QACs: The Development
of Multicationic Quaternary Ammonium Compounds with a High Degree
of Alkyl Chain Substitution. ChemMedChem.

[41] Asante J. Y., Casey C. M., Bezold E. L., Fernando A., McDonough D., Wuest W. M., Minbiole K. P. C. (2025). Resorcinol-based
Bolaamphiphilic
Quaternary Ammonium Compounds. ChemMedChem.

[100] Leitgeb A. J., Feliciano J. A., Sanchez H. A., Allen R. A., Morrison K. R., Sommers K. J., Carden R. G., Wuest W. M., Minbiole K. P. C. (2020). Further Investigations
into Rigidity-Activity Relationships
in BisQAC Amphiphilic Antiseptics. ChemMedChem.

[101] Michaud M. E., Allen R. A., Morrison-Lewis K. R., Sanchez C. A., Minbiole K. P., Post S. J., Wuest W. M. (2022). Quaternary
phosphonium compound unveiled as a potent disinfectant against highly
resistant Acinetobacter baumannii clinical isolates. ACS Infect. Dis..

[42] Wimley W. C. (2010). Describing
the mechanism of antimicrobial peptide action with the interfacial
activity model. ACS Chem. Biol..

[43] Huan Y., Kong Q., Mou H., Yi H. (2020). Antimicrobial peptides:
classification, design, application and research progress in multiple
fields. Front. Microbiol..

[44] Ebbensgaard A., Mordhorst H., Overgaard M. T., Nielsen C. G., Aarestrup F. M., Hansen E. B. (2015). Comparative evaluation of the antimicrobial activity
of different antimicrobial peptides against a range of pathogenic
bacteria. PLoS One.

[45] Geitani R., Ayoub Moubareck C., Touqui L., Karam Sarkis D. (2019). Cationic antimicrobial
peptides: alternatives and/or adjuvants to antibiotics active against
methicillin-resistant Staphylococcus aureus and multidrug-resistant
Pseudomonas aeruginosa. BMC Microbiol..

[46] Chen Y., Vasil A. I., Rehaume L., Mant C. T., Burns J. L., Vasil M. L., Hancock R. E., Hodges R. S. (2006). Comparison
of biophysical
and biologic properties of α-helical enantiomeric antimicrobial
peptides. Chem. Biol. Drug Des..

[47] Szymczak P., Możejko M., Grzegorzek T., Jurczak R., Bauer M., Neubauer D., Sikora K., Michalski M., Sroka J., Setny P., Kamysz W., Szczurek E. (2023). Discovering
highly potent antimicrobial peptides with deep generative model HydrAMP. Nat. Commun..

[48] Wang J., Feng J., Kang Y., Pan P., Ge J., Wang Y., Wang M., Wu Z., Zhang X., Yu J., Zhang X., Wang T., Wen L., Yan G., Deng Y., Shi H., Hsieh C. Y., Jiang Z., Hou T. (2025). Discovery of antimicrobial peptides
with notable antibacterial potency
by an LLM-based foundation model. Sci. Adv..

[49] Jenssen H., Hamill P., Hancock R. E. (2006). Peptide
antimicrobial agents. Clin. Microbiol. Rev..

[50] Tischer M., Pradel G., Ohlsen K., Holzgrabe U. (2012). Quaternary
ammonium salts and their antimicrobial potential: targets or nonspecific
interactions?. ChemMedChem..

[51] Raetz C. R., Whitfield C. (2002). Lipopolysaccharide
endotoxins. Annual review of biochemistry.

[52] DeMars Z., Singh V. K., Bose J. L. (2020). Exogenous fatty acids remodel Staphylococcus
aureus lipid composition through fatty acid kinase. J. Bacteriol..

[53] Hilton K. L., Manwani C., Boles J. E., White L. J., Ozturk S., Garrett M. D., Hiscock J. R. (2021). The phospholipid membrane compositions
of bacterial cells, cancer cell lines and biological samples from
cancer patients. Chemical Science.

[54] Marcotte L., Barbeau J., Edwards K., Karlsson G., Lafleur M. (2005). Influence
of the lipid composition on the membrane affinity, and the membrane-perturbing
ability of cetylpyridinium chloride. Colloids
Surf., A.

[55] Denyer S. P. (1995). Mechanisms
of action of antibacterial biocides. International
biodeterioration & biodegradation.

[56] Vargas-Cuebas G. G., Sanchez C. A., Brayton S. R., Nikoloff A., Masters R., Minbiole K. P. C., Wuest W. M. (2024). Exploring
the Correlation of Dynamic
Surface Tension with Antimicrobial Activities of Quaternary Ammonium-Based
Disinfectants. ChemMedChem.

[57] Sæbø I., Bjørås M., Franzyk H., Helgesen E., Booth J. (2023). Optimization
of the Hemolysis Assay for the Assessment of Cytotoxicity. Int. J. Mol. Sci..

[58] Bobic L., Harbolic A., Warner G. R. (2024). Reproductive
& developmental
toxicity of quaternary ammonium compounds. Biol.
Reprod..

[59] Bodor N., Buchwald P. (2000). Soft drug design: general principles and recent applications. Medicinal research reviews.

